# Determining Sex-Specific Gene Expression Differences in Human Chorion Trophoblast Cells

**DOI:** 10.3390/ijms26052239

**Published:** 2025-03-02

**Authors:** Daphne D. Arena Goncharov, Ryan C. V. Lintao, Rheanna Urrabaz-Garza, Enkhtuya Radnaa, Ananth K. Kammala, Lauren S. Richardson, Ramkumar Menon

**Affiliations:** 1 Division of Basic Science and Translational Research, The University of Texas Medical Branch, Galveston, TX 77555, USArvlintao@up.edu.ph (R.C.V.L.);; 2 Institute of Reproductive Health, National Institutes of Health, University of the Philippines Manila, 623 Pedro Gil Street, Ermita, Manila 1000, Philippines

**Keywords:** fetal membranes, male, female, infection, chorion

## Abstract

Differences in male (M) and female (F) neonates’ premature birth outcomes and placental trophoblast inflammation have been observed but are unknown to occur within the fetal membrane trophoblast layer (chorion trophoblasts [CTC]). This study examined whether sex-based differences in gene expression and inflammatory marker expression can be observed in CTCs under control or infectious inflammatory conditions modeling preterm birth. CTCs from six different patient-derived fetal membrane samples (3M/3F) were cultured and divided into experimental (Lipopolysaccharide [LPS]) and control groups for 6, 12, or 24 h. RNA from CTCs was subjected to RNA-seq, while cytokine multiplex or ELISA detected pro-/anti-inflammatory cytokines, progesterone, and soluble HLA-G in cell supernatants. CTC-M and CTC-F showed sex, time, and stimulant-dependent differential gene expression profiles. Cytokine analysis demonstrated a significantly lower IL-6 production in control CTC-M than in CTC-F. No sex-dependent responses were observed after LPS treatment regarding cytokines. CTC-M produced significantly lower progesterone than CTC-F. The theories of sexual dimorphism linked to placental inflammation may not extend to CTCs. This study supports that the chorion acts as a “great wall” protecting the fetus by being refractory to insults. Further examination into the weaknesses of the chorion barrier and sex-dependent responses of fetal membranes is needed.

## 1. Introduction

Differences in clinical birth outcomes have consistently been observed between male and female conceptuses, with higher rates of stillbirth, preterm birth, spontaneous abortion, and small for gestational age (SGA) observed in male neonates than in female neonates [1,2,3,4,5]. This increased risk of adverse perinatal outcomes in male fetuses has been partly attributed to the prioritization of fetal growth over investment in placental energy reserves [6,7]. Indeed, sexual dimorphism begins shortly after fertilization and continues throughout and beyond prenatal development [8].

Such sexual dimorphism appears to extend to the placenta, one of the feto-maternal interfaces during pregnancy. As an extraembryonic tissue, the placenta reflects the genotypic sex of the fetus [9]. Sex-specific hormonal differences are particularly drastic in the placenta where females prioritize glucocorticoid-mediated signaling to enhance placental energy reserves, while males prioritize androgen-mediated signaling for growth [7]. There were also differences in placental immune response, with a more immunoregulatory response seen in female fetuses compared to male fetuses [8].

However, the placenta–decidual interface is only one of the two existing maternal interfaces, with the other being the chorio-decidual interface at the fetal membranes. The fetal membranes are composed of an inner layer of amnion epithelial cells in contact with the amniotic fluid and an outer layer of chorion trophoblast cells (CTCs) connected by multiple layers of collagen-rich extracellular matrix [10]. Opposite the chorion layer is the maternal immune cell-enriched decidua parietalis. While recent studies have shown that fetal membranes have endocrine [11,12], metabolic [13], transport [14,15,16,17,18] anti-microbial [19], and immune [20,21] functions, in addition to mechanical support, there is little evidence pointing to sex-specific differences in these functions. Sexual dimorphism has been observed in placental trophoblasts from the first trimester [22] up to term [23]. However, such sex-specific differences have not been described in CTCs in the fetal membranes.

Since we recently characterized CTCs as a distinct cell type from placental trophoblasts [24], we investigated in this study whether there is evidence for sexual dimorphism in CTCs. We also determined if differential responses exist between male and female CTCs when they are treated with lipopolysaccharide (LPS), mimicking ascending infection leading to infectious inflammation, which is associated with chorioamnionitis.

## 2. Results

### 2.1. Confirmation of Genotypic Sex of CTCs

**Figure 1:**
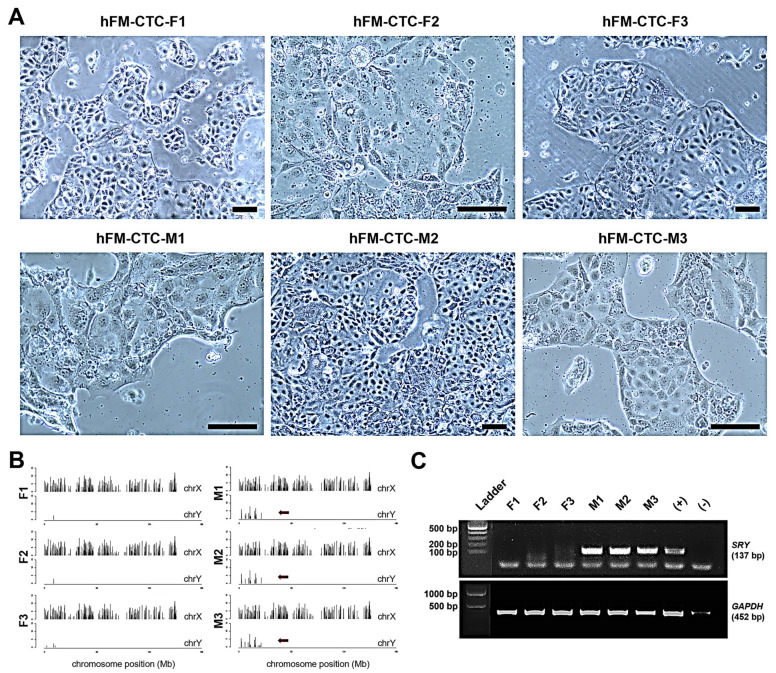
Confirmation of genotypic sex of CTCs. (**A**) Phase-contrast images of three biological replicates of CTCs from female and male placentas. Scale bar, 100 µm. (**B**) Localization of mRNAs in X and Y chromosomes of female and male CTCs. X-axis represents chromosome position, while Y-axis represents median of read density. The red arrow indicates the Y chromosome. (**C**) Electrophoresis gel profile of *SRY* gene amplicon (137 bp) in female and male CTCs, with GAPDH (452 bp) as control.

Figure 1A shows the phase-contrast images of male and female CTCs after immortalization. Overall, the CTCs exhibited a cuboidal, epithelioid phenotype, with male and female CTCs appearing indistinguishable. To confirm the genotypic sex of CTCs, RNA sequencing was done, which shows mRNAs localized in the Y chromosome in male CTCs (Figure 1B). This was further validated via PCR, which shows the presence of the sex-determining region *SRY* gene in male CTCs and its absence in female CTCs (Figure 1C). Therefore, it confirms the isolation of three male and three female CTC cell lines for future experimentation.

### 2.2. Baseline Sex-Specific Differences in CTC Gene Expression

**Figure 2:**
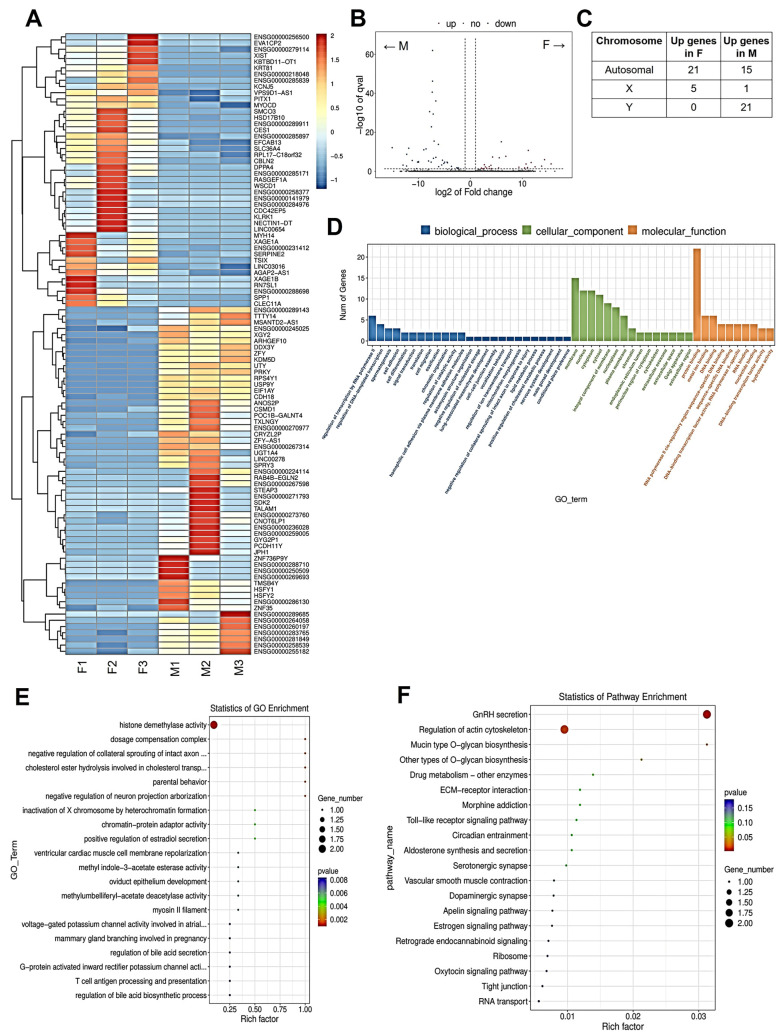
Baseline gene expression differences between female and male CTCs. (**A**) Heatmap and hierarchical clustering of top differentially expressed genes in female and male CTCs. Upregulated genes are represented in red, and downregulated genes are represented in blue. (**B**) Volcano plots of differentially expressed genes between female and male CTCs. Genes significantly enriched in female CTCs are shown in red dots, while genes significantly enriched in male CTCs are shown in blue dots. (**C**) Location of differentially expressed genes between female and male CTCs with respect to human genome. (**D**) Bar graph of gene ontology enrichment analysis. Y-axis indicates the number of genes overlapping the GO term. (**E**) Dot plot of gene ontology (GO) enrichment analysis. Diameter indicates the number of genes overlapping the GO term. Color indicates the enrichment *p*-value. (**F**) Dot plot of Kyoto Encyclopedia of Genes and Genomes (KEGG) enrichment analysis. Diameter indicates the number of genes overlapping the KEGG term. Color indicates the enrichment *p*-value.

To determine baseline differences in gene expression between male and female CTCs, RNA sequencing was done. As shown in the heatmap in Figure 2A, there was an observed heterogeneity in gene expression within male CTCs and female CTCs. Overall, there were 26 upregulated genes and 37 downregulated genes in female CTCs compared to male CTCs (Figure 2B). Of the 63 differentially expressed genes, 36 were autosomal, 6 were X-linked, and 21 were Y-linked (Figure 2C). As expected, all 21 Y-linked genes were upregulated in male CTCs compared to female CTCs. Of the differentially expressed X-linked genes, five were upregulated in female CTCs, while one (*SPRY*) was upregulated in male CTCs. The top GO biological processes were related to transcription regulation, while the top GO molecular functions were related to protein and DNA binding (Figure 2D), suggesting sex-specific differences in gene expression regulation. Of interest, the enriched GO terms were related to chromatin remodeling, such as histone demethylase activity (*KDM5D*, *UTY*) and X chromosome inactivation (*XIST*, *TSIX*), as well as sterol metabolic processes (*CES1*, *SPP1*) (Figure 2E). Looking at KEGG enrichment (Figure 2F), the significantly enriched pathways were gonadotropin-releasing hormone (GnRH) secretion, regulation of actin cytoskeleton, the mucin type, and other types of O-glycan biosynthesis.

### 2.3. Sex-Specific Differences in CTCs in Response to LPS

**Figure 3:**
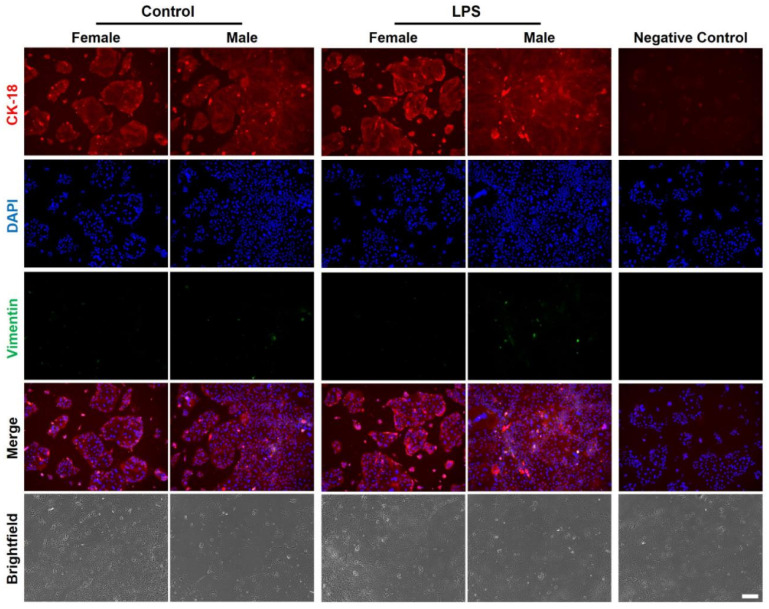
Differences in intermediate filament expression and cellular morphology between male and female CTCs at baseline and in response to LPS. Immunostaining of CK-18 (red) and vimentin (green) after 24 h treatment, with DAPI as nuclear stain. Merged immunofluorescence and brightfield images are also shown. Scale bar, 200 µm.

Next, cells were treated with LPS to determine sex-specific responses to infectious inflammation. Since CTCs, like amnion epithelial cells, can undergo epithelial–mesenchymal transition [25], we looked at cytokeratin-18 (CK-18) and vimentin expression in both sexes via immunostaining. As shown in Figure 3, in control conditions, both female and male CTCs stained predominantly CK-18 with minimal vimentin. However, in response to LPS, they maintained their epithelioid shape and remained predominantly CK-18^+^. No significant difference in overall shape and CK-18 expression was observed between female and male CTCs.

**Figure 4:**
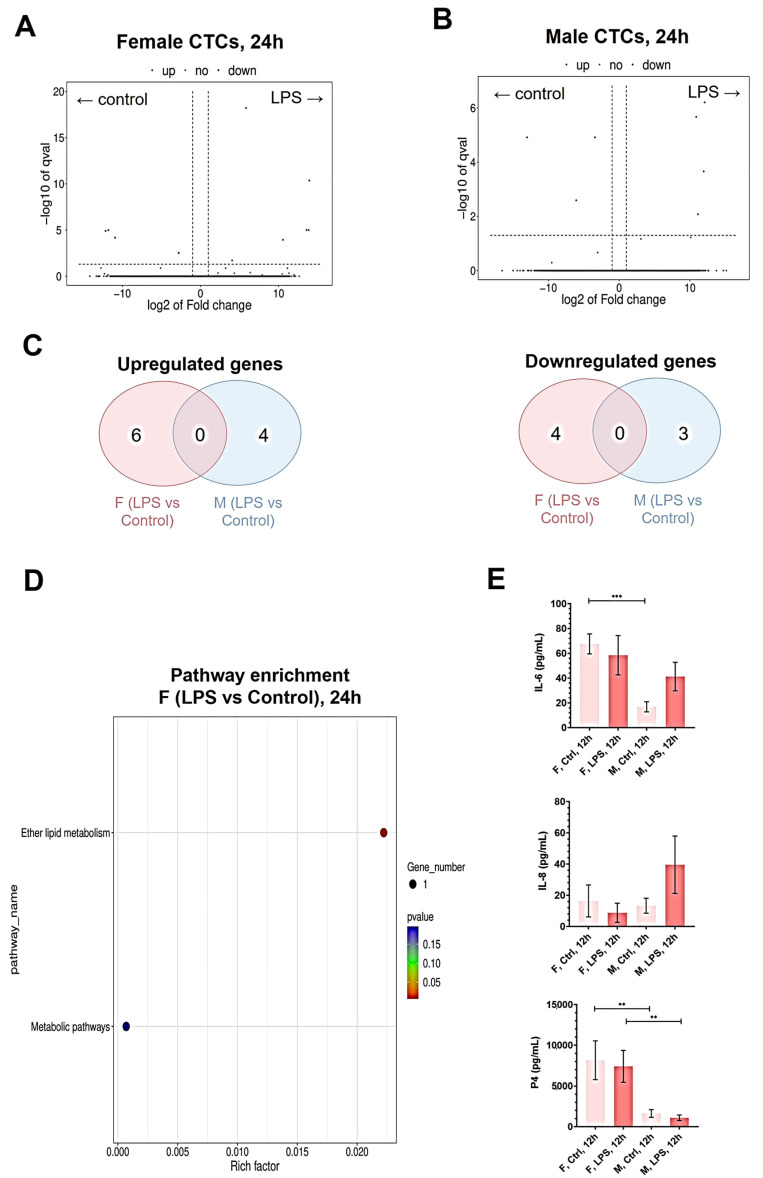
Differential transcriptomic and cellular responses of female and male CTCs to LPS. (**A**,**B**) Volcano plots of differentially expressed genes in (**A**) female and (**B**) male CTCs in response to LPS. Significantly upregulated genes in response to LPS are shown in red dots, while significantly downregulated genes are shown in blue dots. (**C**) Venn diagram of common differentially expressed genes between female and male CTCs. (**D**) Dot plot of Kyoto Encyclopedia of Genes and Genomes (KEGG) enrichment analysis for female CTCs. Diameter indicates the number of genes overlapping the KEGG term. Color indicates the enrichment *p*-value. (**E**) Measurement of IL-6, IL-8, and progesterone production in female and male CTCs at baseline and in response to LPS. Data are presented as mean ± standard error of the mean (SEM). *p* values: **, *p* < 0.01; ***, *p* < 0.001; ns, not significant.

Differential gene expression was also determined in control conditions and in response to LPS treatment after 24 h. Directly comparing female and male CTCs after LPS treatment for 24 h, there were 27 enriched genes in female CTCs and 29 enriched genes in male CTCs. To highlight which genes were in response to LPS, we looked at the differentially expressed genes between control and LPS treatment groups for both sexes. In response to LPS, there were six upregulated and four downregulated genes in female CTCs (Figure 4A), while there were four upregulated genes and three downregulated genes in male CTCs (Figure 4B). These genes were all located in autosomes. As shown in Figure 4C, there was no overlap in differentially expressed genes between female and male CTCs. Pathway analysis showed significant enrichment of only one pathway (ether lipid metabolism) in female CTCs in response to LPS (Figure 4D). No enriched pathways were seen in male CTCs in response to LPS.

In addition to sex-specific transcriptomic responses, we also looked at the production of important soluble mediators by CTCs such as IL-6, IL-8, and progesterone [26] (Figure 4E). In control conditions, female CTCs produce more IL-6 (Figure 4E) and progesterone (Figure 4) than male CTCs, while no significant difference was seen with IL-8 (Figure 4). Interestingly, there were no significant differences in IL-6, IL-8, and progesterone in response to LPS regardless of sex. However, when comparing female and male CTCs after treatment with LPS, there was only an observed significant difference in progesterone levels, although a three-fold increase in IL-8 was observed in male CTCs than in female CTCs.

## 3. Discussion

Sex-specific differences in human placental transcriptome have been reported during early gestation and have been observed to contribute to sex-specific differences in fetal growth trajectories and outcomes later in the second and third trimesters [7]. However, most of these findings came from analyzing chorionic placenta villi samples [23,27,28] and, thus, may not necessarily be reflected in fetal membranes [29]. As sexual dimorphism was reported in placental trophoblasts [22], we wanted to see if this phenomenon extends to chorion trophoblasts. Our study shows the following: (1) CTCs, as part of extraembryonic tissue, exhibit the same genotypic sex as the conceptus, (2) female and male CTCs exhibit very few sex-specific differential gene expression, (3) the few differentially expressed genes between female and male CTCs were on chromatin regulation and metabolic processes, and (4) regardless of sex, CTCs do not exhibit significant transcriptomic or effector changes in response to LPS except for the enrichment of lipid metabolism in female CTCs.

In first-trimester placental trophoblasts, male cells were found to have enriched genes involved in protein translation and mitochondrial and ribosomal functions, while female cells had enriched genes involved in immune function and response to stimuli [22]. This finding is consistent with the prioritization of fetal growth by male fetuses. These sex-specific differences in placental trophoblasts extend up to term, with the enrichment of pathways for graft-versus-host disease and other inflammatory pathways, suggesting an inflammatory response that parallels adverse outcomes in male fetuses [23].

However, the transcriptomic differences in female and male CTCs in this study were found to be not as drastic as those observed in placental trophoblasts. The enrichment of gene ontology terms involved in chromatin remodeling such as histone modifications and dosage compensation complex are important in X chromosome inactivation in females, one of the contributors to sexual dimorphism in pregnancy, along with genomic imprinting, androgen synthesis, and secretion [8]. On the other hand, the enrichment of the GnRH secretion pathway further supports the sex-specific hormonal differences observed in the placental unit, with female fetuses prioritizing glucocorticoid pathways to enhance placental energy reserves, and male fetuses prioritizing androgen pathways to promote growth [7]. This is further supported by the enrichment of the regulation of actin cytoskeleton and mucin biosynthesis in females, suggesting metabolic regulatory processes happening in female CTCs. While the extent of the contribution of the fetal membranes in this remains to be explored, it is possible that the thin architecture and relatively low biomass of fetal membranes, compared to the placenta, may be inefficient as placental reserves [6] that can be modulated by the growing fetus, thus resulting in minimal sex-specific differences in CTCs compared to placental trophoblasts. Additionally, the observation of increased ether lipid metabolism in female CTCs should be investigated to determine the biological relevance of this unique signaling in females versus males.

Interestingly, regardless of sex, CTCs appear to be refractory to LPS, unlike placental trophoblasts [30]. This, along with the resistance of CTCs to the epithelial–mesenchymal transition [25,31], suggests that the chorion acts as a “great wall”, which protects the fetus from external infection. This notion is supported by single-cell RNA profiling of CTCs during the second trimester, which showed a transcriptional program consistent with its role in defense against insults [32]. It is possible that this defensive role is important to be maintained regardless of sex.

While theories of sexual dimorphism in placentas do not appear to extend to CTCs, the main limitation of this study is that it analyzes CTCs removed from their context in the fetal membranes and only uses three biological replicates from each fetal sex. Single-cell transcriptomic profiling of the placental-decidual interface during the late first trimester has identified 91 sexually dimorphic receptor–ligand interactions, which is evidence of maternal–fetal cross-talk [22]. This can also be reflected within in vitro models that allow cellular compartmentalization while maintaining intercellular interactions, such as the use of microphysiological systems, colloquially known as organ-on-chips [26,33]. By employing single-cell transcriptomics in fetal membranes and modeling sexual dimorphism using fetal membrane-on-chip, we can further discern these sexually dimorphic interactions, in addition to the inherent sexual dimorphic characteristics of each cell type.

## 4. Methods

### 4.1. Ethics Statement

Any information collected is in accordance with existing state and federal laws, regulations, and protocols governing health data privacy in the United States. Subjects were recruited and consent was gathered for this study based on the Institutional Review Board of The University of Texas Medical Branch at Galveston, TX, USA (IRB 23-0079, date: 28 March 2024).

### 4.2. Isolation and Immortalization of Chorion Trophoblasts from Male and Female Placentas

Primary chorion trophoblast cells were isolated from term caesarean section fetal membranes of three male and three female offspring, as described by Menon et al. (2020) [34], with modifications from Radnaa et al. (2022) [35]. In summary, the chorion was separated from the amnion and the adherent decidua and blood clots were gently removed with scalpel and washed with prewarmed saline. The chorion tissue was cut into pieces and treated twice with 2.4 U/mL of dispase (Sigma–Aldrich, Burlington, MA, USA) in Hank’s Balanced Salt Solution (HBSS) for 8 min. After rinsing with Dulbecco’s Modified Eagle Medium (DMEM)/F-12 supplemented with 1% penicillin-streptomycin, 1% amphotericin B, and 10% heat inactivated-fetal bovine serum (FBS), the tissue pieces were then incubated with digest buffer-I (0.75 mg/mL collagenase (Sigma–Aldrich, Burlington, MA, USA) and 25 µg/mL of DNase-I (Sigma–Aldrich, Burlington, MA, USA) in HBSS) for 3 h at 37 °C. The undigested tissues were then treated with digest buffer-II (0.25% trypsin and 25 µg/mL of DNase-I (Sigma–Aldrich, Burlington, MA, USA) in phosphate-buffered saline (PBS)) for 5 min at 37 °C. After vortexing, the tissue was filtered through a 70 µm cell trainer, and the resulting cell mixture was centrifuged at 300 *g* for 10 min. The cell pellet was resuspended and maintained in complete CTC media (DMEM/F-12 supplemented with 1% penicillin-streptomycin, 1% amphotericin B, 0.2% heat inactivated-FBS, 0.3% bovine serum albumin, 1X insulin–transferrin–selenium–ethanolamine, 0.8 mM of valproic acid, 0.01 mM of β-mercaptoethanol, 5 µM of Y27632, 2 µM of CHIR99021, 1 µM of SB431542, 0.5 µM of A83-01, 1.5 µg/mL of L-ascorbic acid, and 10 µg/mL of epithelial growth factor). Primary chorion trophoblasts were then immortalized with large T antigen-producing lentivirus SV40T (Cat# CILV01, ALSTEM, Richmond, CA, USA), also as described by Radnaa et al. (2022) [35]. Briefly, 300,000 cells were infected with 10 μL of retroviral supernatant and 4 μL of TransPlus reagent (Cat# V050, ALSTEM, Richmond, CA, USA) in complete CTC media. Selection with 1 μg/mL of puromycin (Cat# P8833, Sigma-Aldrich, Burlington, MA, USA) was performed 72 h post-infection for 7 days. When 90% confluence was reached, CTCs were trypsinized for 10 min in a 37 °C, 5% CO_2_ incubator. Trypsin was inactivated with DMEM containing 10% FBS, and the cells were collected via centrifugation at 300× *g* for 5 min at 20 °C. After removing the supernatant, the cell pellet was resuspended in complete CTC media and was split 1:5. Cell media were changed every 2 days, and cell cultures were maintained at 37 °C, 5% CO_2_ in the Menon Laboratory, The University of Texas Medical Branch at Galveston, TX, USA.

### 4.3. SRY Gene Detection via Gel Electrophoresis

To confirm the male origin of three of our CTC cell lines, a polymerase chain reaction (PCR) was performed for the SRY gene in the Y chromosome. RNA was extracted from isolated fetal membrane leukocytes using TRIzol^®^ reagent (Invitrogen, Life Technologie, Carlsbad, CA, USA), followed by chloroform (Fisher Scientific) and isopropanol (Acros Organics, Geel, Belgium) treatments, to purify total RNA. Total RNA was reverse-transcribed into complementary DNA (cDNA) with a high-capacity RNA-to-cDNA kit (Applied Biosystems, Carlsbad, CA, USA). cDNA was then amplified by PCR using RED Taq^®^ Ready MixTM PCR reaction mix (Sigma, Saint Louis, MO, USA) with the specific primers (for SRY gene, forward: 5′-TGGCGATTAAGTCAAATTCGC-3′ and reverse: 5′-CCCTAGTACCCTGACAATGTATT-3′; for GAPDH gene, forward: 5′-ACCACAGTCCATGCCATCAC-3′ and reverse: 5′-TCCACCACCCTGTTGCTGTA-3′) according to the manufacturer’s instruction. The PCR amplification was conducted using an iCycler thermal cycler (Bio-Rad) under the following conditions: 5 min at 94 °C denaturation followed by 40 cycles at 94 °C for 30 s, 58 °C for 45 s, and 72 °C for 45 s. The PCR products (137 bp for SRY, 452 bp for GAPDH) were then analyzed with 1.5% agarose gel electrophoresis.

### 4.4. Treatment of CTCs with LPS

Stock LPS solution was prepared by dissolving 1 mg of lipopolysaccharides from *Escherichia coli* O55:B5 (Sigma–Aldrich, Burlington, MA, USA) in 1 mL of ultrapure water and stored at –20 °C. CTCs (300,000 cells/well) were seeded in 6-well plates and were allowed to attach overnight at 37 °C, 5% CO_2_. The following day, the cell media were removed, and the CTCs were treated with 100 ng/mL of LPS to induce infectious inflammation for 12 and 24 h. Complete CTC media served as control. Three male and three female biological replicates per treatment and per duration were prepared.

### 4.5. Immunofluorescence Staining of CTCs

After 24 h treatment duration, adherent cells were washed with PBS before fixing with 4% paraformaldehyde for 20 min. The cells were permeabilized with 0.5% Triton-X in 1× PBS for 10 min and blocked with 3% BSA in 1× PBS for 30 min. The cells were incubated overnight with primary antibodies CK-18 (mouse 1:300; Cat# ab668, Abcam, Cambridge, UK) and vimentin (rabbit, 1:300; Cat# ab92547, Abcam, Cambridge, UK). The next day, the cells were washed with 1× PBS and incubated for 1 h in the dark with secondary antibodies AlexaFluor™ 594-conjugated goat-anti mouse (1:1000; ThermoFisher Scientific, Waltham, MA, USA) and AlexaFluor™ 488-conjugated donkey anti-rabbit (1:1000; Abcam, Cambridge, UK) diluted in 1× PBS. The cells were washed with 1× PBS and stained with NucBlue Fixed ReadyProbes reagent (ThermoFisher Scientific, Waltham, MA, USA) for 5 min. After a final round of washing, the cells were mounted in Mowiol 4–88 mounting medium (Sigma–Aldrich, Burlington, MA, USA) and viewed under Keyence BZ-X810 microscope (Keyence, Itasca, IL, USA).

### 4.6. Luminex IL-6 and IL-8 Detection

To detect IL-6 and IL-8, Custom MILLIPLEX^®^ Human Cytokine/Chemokine/Growth Factor Panel A kit (Cat# HCYTA-60K-06; EMD Millipore, Burlington, MA, USA) was performed following manufacturer’s instructions. In summary, a combination of cell culture media obtained from 6-well plates at 12-h timepoint, assay buffer, and magnetic beads were placed in each well for overnight incubation. Detection antibodies and streptavidin-phycoerythrin were subsequently added to each well, and following multiple rounds of incubation and washing, the magnetic beads were resuspended in xMAP^®^ Sheath Fluid PLUS (MilliporeSigma, Burlington, MA, USA). The plate was then run on Luminex^®^ 200^TM^ instrument (Austin, TX, USA).

### 4.7. Competitive Progesterone Enzyme-Linked Immunosorbent Assay (ELISA)

Progesterone competitive ELISA kit (Cat# EIAP4C21, Thermo Fisher Scientific, Waltham, MA, USA) was performed following manufacturer’s instructions. In summary, 1:5 dilution of cell culture media obtained from 6-well plates at 12-h timepoint was combined with peroxidase-conjugated progesterone and anti-progesterone antibody in each IgG-coated well. After incubation and washing, a colorimetric substrate was added to each well. Absorbance was measured at 450 nm using a microplate reader.

### 4.8. RNA Extraction from CTCs for RNAseq

At 24-h timepoint, the cells were disrupted from the plates with a cell scraper. To isolate RNA, Monarch Total RNA Miniprep Kit (Cat# T2010S, New England Biolabs, Ipswich, MA, USA) was performed following manufacturer’s instructions. Briefly, RNA lysis buffer was added to each sample. Subsequently, gDNA removal was performed through a series of centrifugation steps using gDNA removal columns. Remaining sample was then mixed with an equal volume of 95% ethanol, and additional centrifugation steps were performed in RNA purification columns. RNA was collected via a final centrifugation step in nuclease-free water. RNA yield and purity were measured using NanoDrop™ Eight Spectrophotometer (ThermoFisher Scientific, Waltham, MA, USA). The samples were then stored at –80 °C until they were sent for analysis.

### 4.9. RNA Library Construction and Sequencing

Library construction and RNA sequencing were performed by LC Sciences (Houston, TX, USA). The total RNA quantity and purity were measured by Bioanalyzer 2100 and RNA 6000 Nano LabChip Kit (Cat# 5067-1511, Agilent, Santa Clara, CA, USA), and high-quality RNA samples with RNA integrity number (RIN) > 7.0 were used to construct sequencing library. mRNA was purified from total RNA (5 µg) using Dynabeads™ Oligo(dT)_25_ (Thermo Fisher Scientific, Waltham, MA, USA) with two rounds of purification. Following purification, the mRNA was fragmented into short fragments using divalent cations under elevated temperature using NEBNext^®^ Magnesium RNA Fragmentation Module (Cat# e6150, New England Biolabs, Ipswich, MA, USA) under 94 °C for 5–7min. Then the cleaved RNA fragments were reverse transcribed to create the cDNA by SuperScript™ II Reverse Transcriptase (Cat# 1896649, Thermo Fisher Scientific, Waltham, MA, USA), which were next used to synthesise U-labeled second-stranded DNAs with E. coli DNA polymerase I (Cat# m0209, New England Biolabs, Ipswich, MA, USA), RNase H (Cat# m0297, New England Biolabs, Ipswich, MA, USA), and dUTP Solution (Cat# R0133, Thermo Fisher Scientific, Waltham, MA, USA). An A-base was then added to the blunt ends of each strand, preparing them for ligation to the indexed adapters. Each adapter contained a T-base overhang for ligating the adapter to the A-tailed fragmented DNA. Dual-index adapters were ligated to the fragments, and size selection was performed with AMPureXP beads. After the heat-labile UDG enzyme (Cat# m0280, New England Biolabs, Ipswich, MA, USA) treatment of the U-labeled second-stranded DNAs, the ligated products were amplified with PCR by the following conditions: initial denaturation at 95 °C for 3 min; 8 cycles of denaturation at 98 °C for 15 sec, annealing at 60 °C for 15 sec, and extension at 72 °C for 30 sec; and then final extension at 72 °C for 5 min. The average insert size for the final cDNA library was 300 ± 50 bp. Following the vendor’s recommended protocol, 2 × 150 bp paired-end sequencing (PE150) was performed on an Illumina Novaseq™ 6000 sequence platform (Illumina, San Diego, CA, USA).

### 4.10. Filtering of Clean Reads and Alignment with Reference Genome

Reads obtained from the sequencing machines include raw reads containing adapters, or low-quality bases that will affect the following assembly and analysis. Thus, to get high-quality clean reads, reads were further filtered by Cutadapt (https://cutadapt.readthedocs.io/en/stable/; accessed on 8 January 2025) [36]. The parameters were as follows: (1) removing reads containing adapters; (2) removing reads containing polyA and polyG; (3) removing reads containing more than 5% of unknown nucleotides (N); and (4) removing low-quality reads containing more than 20% of low-quality (Q-value ≤ 20) bases. Then, sequence quality was verified using FastQC (http://www.bioinformatics.babraham.ac.uk/projects/fastqc/; accessed on 8 January 2025), including the Q20, Q30, and GC content of the clean data. The reads of all samples were aligned to the human reference genome using HISAT2 (https://daehwankimlab.github.io/hisat2/; accessed on 8 January 2025) package [37], which initially removed a portion of the reads based on quality information accompanying each read and then mapped the reads to the reference genome.

### 4.11. Quantification of Gene Abundance

The mapped reads of each sample were assembled using StringTie (http://ccb.jhu.edu/software/stringtie/, accessed on 8 January 2025, v1.3.4d) with default parameters [38]. Then, all transcriptomes from all samples were merged to reconstruct a comprehensive transcriptome using gffcompare software (http://ccb.jhu.edu/software/stringtie/gffcompare.shtml, accessed on 8 January 2025, v0.9.8). After the final transcriptome was generated, StringTie and ballgown (https://www.bioconductor.org/packages/release/bioc/html/ballgown.html; accessed on 8 January 2025) were used to estimate the expression levels of all transcripts and perform expression abundance for mRNAs by calculating FPKM (fragment per kilobase of transcript per million mapped reads) value.

### 4.12. Differentially Expressed Gene (DEG) Analysis

Gene differential expression analysis was performed by DESeq2 software [39] between two different groups (and by edgeR [40] between two samples). The genes with the parameter of false discovery rate (FDR) below 0.05 and absolute fold change ≥ 2 were considered differentially expressed genes. Differentially expressed genes were then subjected to enrichment analysis of GO functions and KEGG pathways.

### 4.13. Other Bioinformatics Analysis

Principal component analysis (PCA) was performed with CLC Genomics Workbench v23.0.3 (QIAGEN, Hilden, Germany). Log CPM (Counts per Million) values were calculated for each gene. Z-score normalization was performed in the samples for each gene, where the counts for each gene were mean-centered and scaled to unit variance. Genes with no detectable expression in all samples were removed. Gene ontology (GO) and pathway enrichment analyses were performed by LC Sciences (Houston, TX, USA) using in-house Perl scripts. GO terms with *p* < 0.05 were defined as significantly enriched GO terms in DEGs, while pathways with *p* < 0.05 were defined as significantly enriched pathways in DEGs.

### 4.14. Statistical Analysis

Multiple unpaired two-tailed *t*-tests were employed to determine if the differences in cytokine and other soluble mediators between control and experimental treatment conditions were statistically significant. *p*-values of 0.05 or less were considered statistically significant. Data were analyzed and visualized using GraphPad Prism 9.5.0 (GraphPad Software, Boston, MA, USA).

## Data Availability

Targeted RNA-seq data have been deposited at Gene Expression Omnibus under accession number GSE288056 and are publicly available as of publication. No original code is reported in this paper. Any additional information required to reanalyze the data reported in this paper is available from the lead contact upon request.
